# An engineered genetic selection for ternary protein complexes inspired by a natural three-component hitchhiker mechanism

**DOI:** 10.1038/srep07570

**Published:** 2014-12-22

**Authors:** Hyeon-Cheol Lee, Alyse D. Portnoff, Mark A. Rocco, Matthew P. DeLisa

**Affiliations:** 1 School of Chemical and Biomolecular Engineering, Cornell University, Ithaca, NY 14853 USA; 2 Department of Biomedical Engineering, Cornell University, Ithaca, NY 14853 USA

## Abstract

The bacterial twin-arginine translocation (Tat) pathway is well known to translocate correctly folded monomeric and dimeric proteins across the tightly sealed cytoplasmic membrane. We identified a naturally occurring heterotrimer, the *Escherichia coli* aldehyde oxidoreductase PaoABC, that is co-translocated by the Tat translocase according to a ternary “hitchhiker” mechanism. Specifically, the PaoB and PaoC subunits, each devoid of export signals, are escorted to the periplasm in a piggyback fashion by the Tat signal peptide-containing subunit PaoA. Moreover, export of PaoA was blocked when either PaoB or PaoC was absent, revealing a surprising interdependence for export that is not seen for classical secretory proteins. Inspired by this observation, we created a bacterial three-hybrid selection system that links the formation of ternary protein complexes with antibiotic resistance. As proof-of-concept, a bispecific antibody was employed as an adaptor that physically crosslinked one antigen fused to a Tat export signal with a second antigen fused to TEM-1 β-lactamase (Bla). The resulting non-covalent heterotrimer was exported in a Tat-dependent manner, delivering Bla to the periplasm where it hydrolyzed β-lactam antibiotics. Collectively, these results highlight the remarkable flexibility of the Tat system and its potential for studying and engineering ternary protein interactions in living bacteria.

The hallmark of the twin-arginine translocation (Tat) pathway is its unique ability to transport folded proteins across the tightly sealed cytoplasmic membrane (reviewed in ref. 1 and elsewhere). At present, the exact mechanistic details of this process remain poorly understood; however, the versatility of the Tat system is firmly established on the basis of the structural and functional diversity of proteins that transit this pathway. Indeed, Tat substrates range in size between 20 and 70 Å in diameter, but also much smaller in the case of some engineered substrates^2^, and include soluble periplasmic enzymes^3^,^4^,^5^, lipoproteins^6^, and inner and outer membrane proteins^7^,^8^,^9^.

While the bulk of Tat substrates studied so far are monomeric proteins (e.g., *Escherichia coli* molybdoenzyme TorA), heterodimeric proteins can also transit this pathway. One notable example is the nickel-iron [NiFe] hydrogenase 2 (HYD2) system of *E. coli* that catalyzes the reversible oxidation of hydrogen and allows bacteria to use hydrogen as an energy source for growth. HYD2 is a heterodimer comprised of a large subunit (HybC), containing the [NiFe] active site but lacking any discernible export signal, and a small subunit (HybO), bearing iron-sulfur [Fe-S] clusters and also an N-terminal Tat signal peptide. Besides assembling [Fe-S] clusters, the HybO subunit also assembles with the large HybC subunit in the cytoplasm prior to export. Following assembly, the HybOC heterodimer is exported to the periplasm by virtue of the Tat signal peptide on the HybO subunit^9^. This mode of export, whereby one substrate protein devoid of any known export signal is co-translocated in a complex with its signal peptide-bearing partner, is referred to as “hitchhiker” co-translocation^9^.

A handful of additional substrates are predicted to follow the hitchhiker mechanism^10^,^11^,^12^, which has recently inspired new methods for expressing and engineering heterologous proteins. For example, it has been shown that preassembled dimeric proteins, including the covalently linked heavy and light chains of a F_AB_ antibody, can be targeted to the periplasm via the hitchhiker route^13^. More recently, hitchhiker-mimetic genetic assays for monitoring and engineering pairwise protein interactions have been reported^14^,^15^. In these assays, the test protein (i.e., bait or receptor) to be screened is engineered with an N-terminal Tat signal peptide, whereas the known or putative partner protein (e.g., prey or ligand) is fused to a reporter enzyme whose co-translocation to the periplasm gives rise to a distinct and quantifiable phenotype. For example, by using mature TEM1 β-lactamase (Bla) as the reporter enzyme, the binding between a receptor and its ligand can be conveniently linked to antibiotic resistance^15^,^16^.

To find additional examples of hitchhiker substrates that might spawn similar technology development, we turned our attention to the molybdenum-containing iron-sulfur flavoprotein PaoABC (formerly YagTSR) from *E. coli*. PaoABC is an aldehyde oxidoreductase that oxidizes a broad spectrum of aldehydes to their respective acids^17^. The 135-kDa enzyme comprises a non-covalent (αβγ) heterotrimer with a large (78.1 kDa) molybdenum cofactor (Moco)-containing PaoC subunit, a medium (33.9 kDa) flavin adenine dinucleotide (FAD)-containing PaoB subunit, and a small (21.0 kDa) 2 × [2Fe2S]-containing PaoA subunit^18^. Only the PaoA protein contains an N-terminal signal peptide, which can deliver heterologous proteins to the periplasm via the Tat pathway^19^. The fact that only PaoA carries a Tat signal peptide led to an early hypothesis that the heterotrimeric PaoABC complex may be co-translocated^20^; however, experimental evidence in support of this hypothesis has been lacking. Here, we show that PaoABC is a *bona fide* Tat substrate. Moreover, the PaoB and PaoC subunits, which are each devoid of any known export signals, are escorted to the periplasm by PaoA in a piggyback fashion. Akin to HybOC, there is an interdependence between the small, signal-peptide bearing PaoA subunit and the larger PaoB and PaoC subunits for productive membrane translocation of PaoABC.

Drawing inspiration from this three-component hitchhiker mechanism, we developed a genetic selection for studying and engineering ternary protein complexes. Our hypothesis was that a bispecific affinity protein could be used as an adaptor to co-recruit one ligand fused to a Tat export signal and a second ligand fused to a periplasmic reporter protein. To test this notion, a dual antigen-binding protein was created by recombinant fusion of two different single-chain Fv (scFv) antibodies joined by a flexible Gly-Ser linker, thereby yielding a tandem scFv construct. Two additional chimeras were created by fusing the cognate ligand of each scFv to the Tat-dependent signal peptide of *E. coli* TorA and mature Bla, respectively. Co-expression of the three chimeras in *E. coli* resulted in the formation of a non-covalent heterotrimer that was exported in a Tat-dependent manner, thereby delivering Bla to the periplasm where it hydrolyzed β-lactam antibiotics. This new bacterial three-hybrid (B3H) system opens the door to routine selection of ternary protein complexes, especially those involving bispecific affinity proteins, all without the need for purification or immobilization of either binding target.

## Results

### Export of PaoABC is Tat dependent and involves a hitchhiker mechanism

**Figure 1:**
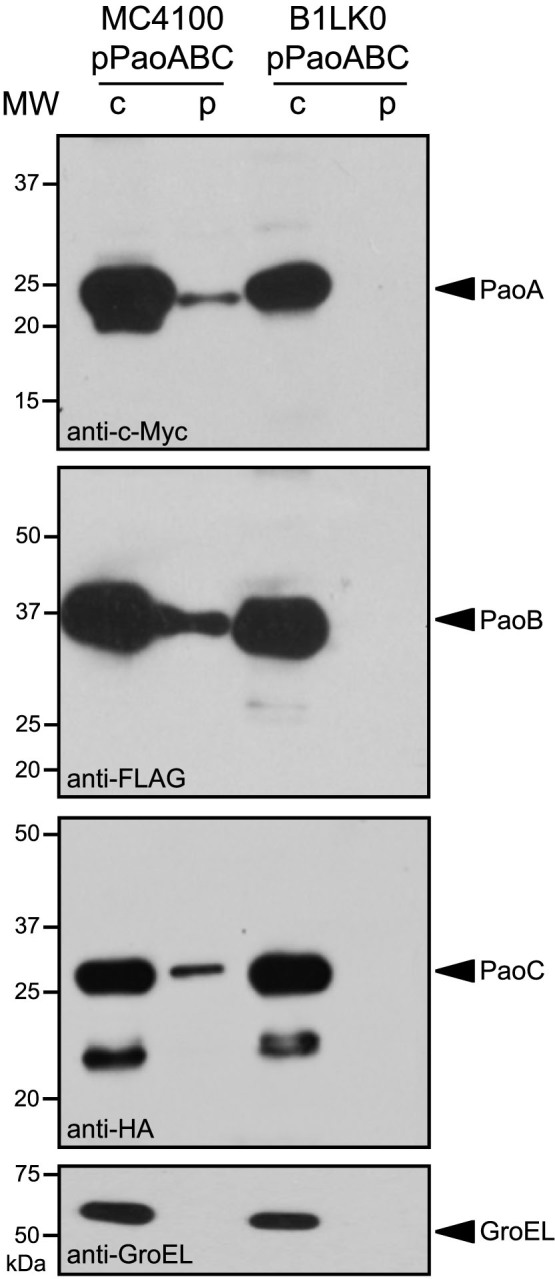
Tat-mediated hitchhiker export of PaoABC heterotrimer. Western blot analysis of cytoplasmic (c) and periplasmic (p) fractions prepared from *E. coli* MC4100 or B1LK0 cells carrying plasmid pPaoABC, which encodes the synthetic *paoABC* operon. Fractions were normalized to an equivalent number of cells prior to loading. Co-expression of epitope-tagged PaoA-c-Myc, PaoB-FLAG and PaoC-HA were detected using anti-c-Myc, anti-FLAG, and anti-HA antibodies, respectively. The cytoplasmic chaperone GroEL was detected using anti-GroEL antibody and served as a fractionation marker. Molecular weight (MW) markers are indicated on the left. See Supplementary Fig. S7 for uncropped versions of the images.

*E. coli* PaoA carries a canonical Tat signal peptide^10^,^19^ and is known to form a heterotrimeric complex with PaoB and PaoC^17^. Hence, we hypothesized that PaoABC might be exported out of the cytoplasm by the Tat translocase according to a hitchhiker mechanism whereby PaoB and PaoC are ferried to the periplasm by the signal peptide-bearing PaoA subunit. To test this notion, the *paoABC* genes were assembled into a synthetic operon under the control of a single Trc promoter in plasmid pTrc99Y using yeast-based recombineering^21^ (Supplementary Fig. S1). The resulting plasmid, pPaoABC, permits co-expression of the PaoABC subunits each appended with a unique C-terminal tag for specific immunodetection. Following expression of the synthetic *paoABC* operon in wild-type (wt) MC4100 cells, all three PaoABC subunits were detected in the periplasm, with a comparable amount of each subunit also detected in the cytoplasm (Fig. 1). In B1LK0 cells, which lack the essential TatC component of the translocase^22^, each PaoABC subunit accumulated only in the cytoplasm (Fig. 1), indicating that transport of PaoABC to the periplasm is dependent on the Tat pathway.

**Figure 2:**
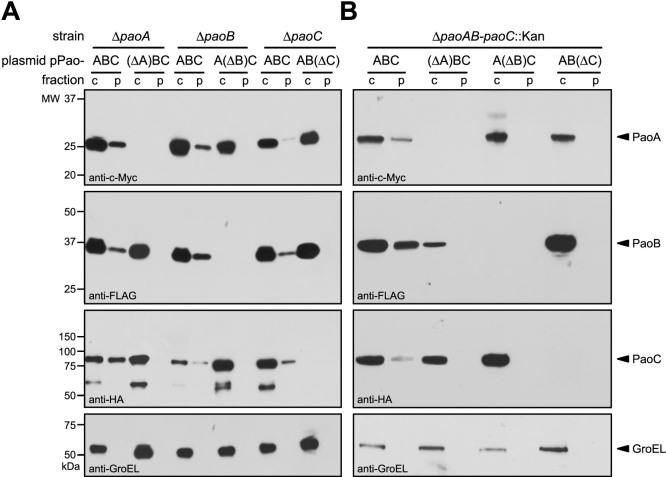
Interdependence of three PaoABC subunits for export. Western blot analysis of cytoplasmic (c) and periplasmic (p) fractions prepared from *E. coli* strain BW25113 and isogenic derivatives lacking individual *pao* genes (A) or the complete *paoABC* operon (B). Strains were complimented with plasmid pPaoABC or derivatives of pPaoABC lacking individual subunits as indicated. Fractions were normalized to an equivalent number of cells prior to loading. Co-expression of epitope-tagged PaoA-c-Myc, PaoB-FLAG and PaoC-HA were detected using anti-c-Myc, anti-FLAG, and anti-HA antibodies, respectively. The cytoplasmic chaperone GroEL was detected using anti-GroEL antibody and served as a fractionation marker. Molecular weight (MW) markers are indicated on the left. See Supplementary Fig. S8 for uncropped versions of the images.

If PaoABC export involves a hitchhiker mechanism, then the absence of PaoA should result in cytoplasmic accumulation of the PaoB and PaoC subunits even in the presence of a functional Tat translocase. This idea was tested by constructing plasmid pPao(ΔA)BC, which encoded the *pao* operon but without the gene encoding PaoA. This plasmid was used to transform Δ*paoA* cells or alternatively HL0281 cells (BW25113 Δ*paoAB-paoC*::Kan) to eliminate possible interference from endogenous Pao proteins. In support of our hitchhiker hypothesis, when signal-peptide bearing PaoA was absent, targeting of PaoB and PaoC to the periplasm was abolished and both proteins accumulated exclusively in the cytoplasm (Fig. 2A and B).

### PaoB and PaoC subunits influence targeting of PaoA

In the case of HybOC export, both HybC and HybO accumulated in the cytoplasm as a precursor when either was expressed in the absence of the other^9^. This interdependence between the small and large subunits for export signified a unique quality control (QC) mechanism not seen for classical secretory proteins, namely that the folding and proper assembly of the subunits is necessary for translocation to the periplasm. To determine if export of PaoABC was governed by a similar QC mechanism, we created plasmids pPaoA(ΔB)C and pPaoAB(ΔC), which lacked the gene encoding the PaoB or PaoC subunit, respectively, and used these to transform the respective Δ*paoB*, Δ*paoC*, or Δ*paoAB-paoC*::Kan strains. When either the PaoB or PaoC subunit was absent, the other two subunits including the signal peptide-bearing PaoA protein accumulated in the cytoplasm (Fig. 2A and B). Therefore, while PaoA is required for membrane targeting of PaoB and PaoC, the presence of the PaoA signal peptide itself is not sufficient for translocation if either of the other subunits is missing, reminiscent of the situation observed for HybOC^9^.

The interdependence among the three subunits highlights the importance of PaoABC heteroassembly in the export process. To confirm complex formation *in vivo* under the conditions used here, we co-produced PaoB and PaoC along with a hexahistidine-tagged version of PaoA in Δ*paoAB-paoC*::Kan cells and subjected soluble lysates derived from these cells to Ni^2+^-affinity chromatography. Immunoblotting revealed that PaoB and PaoC co-eluted with the affinity-purified PaoA (Fig. S2A). In contrast, no Pao proteins were detected in elution fractions prepared from cells co-producing PaoB and PaoC in the absence of PaoA (Fig. S2A). We next subjected the same eluted fractions containing the co-purified proteins to blue native polyacrylamide gel electrophoresis (BN-PAGE), a special case of native electrophoresis for high-resolution separation and molecular mass estimation of native proteins and protein complexes^23^. Two distinct bands were observed: a faint band of ~135 kDa corresponding to the expected PaoABC trimer (Fig. S2B). A second more prominent band of ~270 kDa was also observed (Fig. S2B). While the identity of this larger complex was not confirmed, it may correspond to a possible dimer of PaoABC trimers. In further support of complex formation *in vivo*, gel filtration analysis of the periplasmic fraction derived from Δ*paoAB-paoC*::Kan cells expressing PaoABC revealed a prominent 135-kDa peak corresponding to the PaoABC trimer (Fig. S2C), indicating that complex formation was not an artifact of the Ni^2+^-affinity purification process. When the same periplasmic fraction was subjected to BN-PAGE analysis, we again observed bands corresponding to PaoABC (~135 kDa) and also the unidentified ~270-kDa complex (Fig. S2D). Collectively, these data confirm formation of a stable αβγ heterotrimer *in vivo* that we suspect may further assemble into a (αβγ)_2_ complex.

### A three-hybrid genetic selection inspired by PaoABC co-translocation

**Figure 3:**
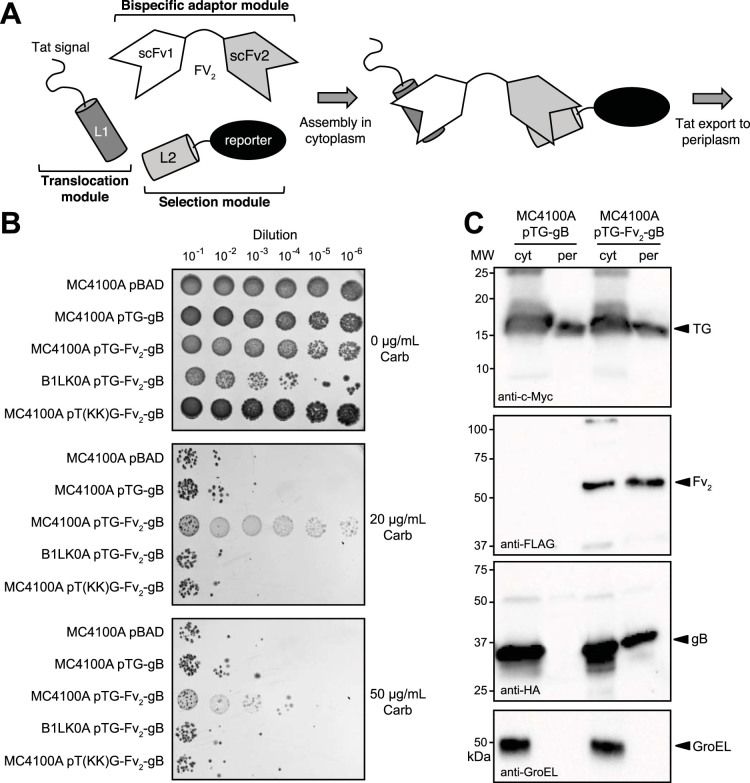
B3H selection system for studying and engineering ternary protein complexes. (A) Schematic of the engineered B3H selection system. The first ligand (L1) modified with a Tat signal peptide serves as the translocation module while the second ligand (L2) fused to the reporter enzyme Bla functions as the selection module. Introduction of a bispecific adaptor module (e.g., bispecific antibody, Fv_2_) creates a physical crosslink between the Tat signal peptide and Bla, thereby resulting in periplasmic accumulation of the ternary complex and conferral of antibiotic resistance to host cells. (B) Selective “spot” plating of serially diluted MC4100A or B1LK0A cells carrying plasmid pTG-Fv_2_-gB encoding B3H components. Additional controls included MC4100A cells carry empty plasmid (pBAD), a plasmid lacking the bispecific Fv_2_ (pTG-gB), or a plasmid encoding a non-functional KK mutant signal peptide (pT(KK)G-Fv_2_-gB). Overnight cultures were serially diluted in liquid LB and spot plated on LB-agar supplemented with Carb (0–50 μg/mL) and arabinose (0.2% w/v). (C) Western blot analysis of cytoplasmic (cyt) and periplasmic (per) fractions prepared from MC4100A cells carrying pTG-gB or pTG-Fv_2_-gB. Fractions were normalized to an equivalent number of cells prior to loading. Blots were probed with anti-c-Myc, anti-FLAG, or anti-HA antibodies to detect each B3H component individually. Quality of the fractions was confirmed by probing membranes with an anti-GroEL antibody. Molecular weight (MW) markers are indicated on the left. See Supplementary Fig. S9 for uncropped versions of the images.

We previously demonstrated that the two-component hitchhiker mechanism governing HybOC export^9^ could be exploited in a general way for monitoring binary protein interactions in *E. coli*^15^,^16^,^24^. Here, we speculated that three-component hitchhiker export by the Tat pathway could be leveraged to create a genetic selection for studying and engineering ternary protein interactions. As proof-of-concept, a bispecific antibody (BsAb) was created by recombinant fusion of two different scFv antibodies joined through a Gly-Ser linker in plasmid pTG-Fv_2_-gB (Fig. S3). This tandem scFv (Fv_2_) design forms the basis of *bi*specific *T*-cell *e*ngager molecules (BiTEs) developed for cancer immunotherapy^25^. We chose two scFv molecules that were previously optimized for intracellular expression because in our system scFv folding and antigen binding must occur cytoplasmically. Specifically, one of the two Fv fragments was directed against the 47-residue basic region-leucine zipper domain of the yeast transcription factor Gcn4 (scFv-GCN4)^15^,^26^ and the other was directed against the capsid protein D (gpD) of bacteriophage lambda (scFv-D10)^27^. The cognate antigens for the engineered Fv_2_ were cloned in the same plasmid (Fig. S3), where each was modified as follows: the Gcn4 leucine zipper domain was modified with an N-terminal Tat signal peptide derived from *E. coli* TorA (TG)^15^ and gpD was fused to the N-terminus of mature Bla lacking its native export signal (gB). Together, these engineered chimeras form the basis of a B3H assay, whereby the BsAb creates a physical crosslink between the Tat signal peptide and the Bla reporter enzyme and the subsequent export of this non-covalent heterotrimer confers resistance to β-lactam antibiotics due to the presence of Bla in the periplasm (Fig. 3A).

### Dual antigen-binding activity is faithfully reported by B3H assay

To evaluate our engineered B3H assay, *E. coli* strain MC4100A was transformed with plasmid pTG-Fv_2_-gB and the resulting cells were challenged with varying concentrations of carbenicillin (Carb). Cells co-producing the TG, Fv_2_, and gB fusions from pTG-Fv_2_-gB showed a much greater resistance to Carb than control cells carrying either an empty plasmid or plasmid pTG-gB, which lacked the Fv_2_ adaptor (Fig. 3B). To confirm whether the resistant phenotype of MC4100A pTG-Fv_2_-gB cells depended on the Tat pathway, we mutated the RR motif in the TorA signal peptide to KK, a substitution known to completely block export^13^. Indeed, cells co-producing T(KK)G, Fv_2_, and gB from pT(KK)G-Fv_2_-gB were sensitive to Carb (Fig. 3B). Likewise, when the original pTG-Fv_2_-gB was used to transform Δ*tatC* mutant cells, the Carb-resistant phenotype was abolished (Fig. 3B). In all cases involving MC4100A cells, plating on non-selective medium resulted in comparable robust growth indicating that the drug sensitivity observed for control cells was not due to inherent growth defects (Fig. 3B). In the case of B1LK0A cells, there was a moderate growth defect observed in the absence of Carb; however, this decreased resistance was not sufficient to account for the near complete inhibition in the presence of 20 and 50 μg/ml Carb. This, in addition to the results with pT(KK)G-Fv_2_-gB in MC4100A cells, suggested that the strong Carb resistance observed for MC4100A pTG-Fv_2_-gB cells depended on the Tat pathway.

Immunoblotting of subcellular fractions prepared from MC4100A pTG-Fv_2_-gB cells confirmed that all three chimeras accumulated in the cytoplasm as intact fusion proteins, albeit with some evidence for degradation (Fig. 3C). Consistent with the resistant phenotype observed above, all three intact fusions were detected in the periplasm of these cells. Since TG was the only fusion carrying a signal peptide, we conclude that export of Fv_2_ and gB must occur by a similar three-component hitchhiker mechanism used by PaoABC. In the absence of the Fv_2_ adaptor, gB was no longer detected in the periplasm (Fig. 3C) confirming that export of the Bla-containing chimera depended on heteroassembly. Not surprisingly, TG still localized in the periplasm even when the adaptor was absent (Fig. 3C), indicating that the subunit interdependence seen above for PaoABC export was not an inbuilt feature of this engineered hitchhiker process. Regardless, these results confirm that our B3H assay reliably detects ternary complex formation in the cytoplasm of living *E. coli* cells.

### Reprogramming antigen specificity by genetic selection of a combinatorial library

**Figure 4:**
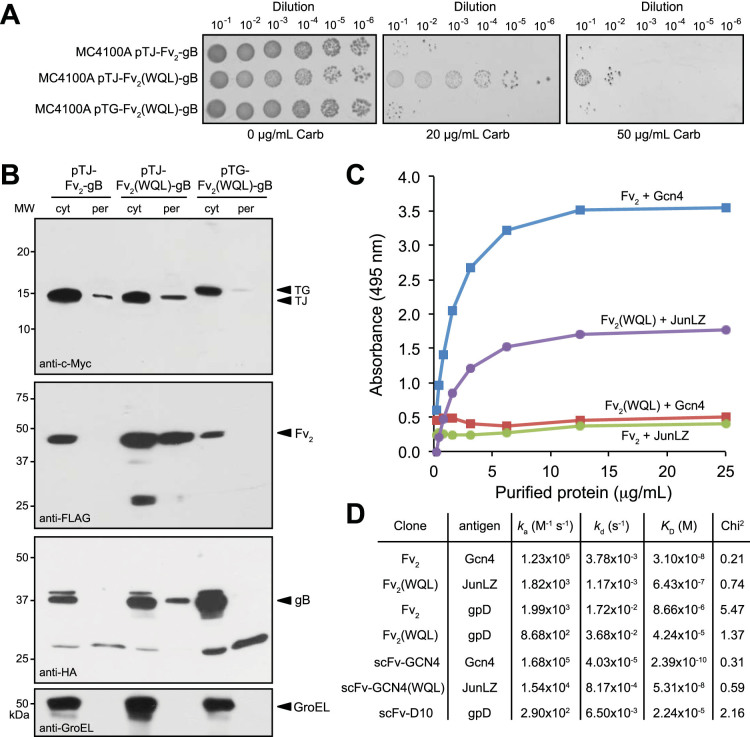
Reprogramming antigen specificity using B3H-mediated library selection. (A) Selective spot plating of serially diluted MC4100A cells carrying pTJ-Fv_2_-gB, pTJ-Fv_2_(WQL)-gB, or pTG-Fv_2_(WQL)-gB. Overnight cultures were serially diluted in liquid LB and spot plated on LB-agar supplemented with Carb (0–50 μg/mL) and arabinose (0.2% w/v). (B) Western blot analysis of cytoplasmic (cyt) and periplasmic (per) fractions prepared from the same cells as in (A). Fractions were normalized to an equivalent number of cells prior to loading. Blots were probed with anti-c-Myc, anti-FLAG, and anti-HA antibodies to detect each component individually. Quality of fractions was confirmed by probing membranes with an anti-GroEL antibody. Molecular weight (MW) markers are indicated on the left. See Supplementary Fig. S10 for uncropped versions of the images. (C) Binding activity of purified Fv_2_ and Fv_2_(WQL) proteins against immobilized MBP-GCN4 and MBP-JunLZ antigens as measured by ELISA. Bound Fv_2_ molecules were detected with anti-His antibodies. (D) Affinity determination of purified bispecific Fv_2_s or unfused scFvs to cognate antigens as measured by surface plasmon resonance (SPR). Cognate antigens were immobilized on CM5 chips and the response of varied amounts of bispecific Fv_2_s or unfused scFvs was compared with an empty flow cell. The binding kinetics of bispecific Fv_2_s or unfused scFvs were monitored using Biacore and affinity values were obtained by fitting the equilibrium binding responses with a 1:1 Langmuir binding model using a simultaneous non-linear program (for sensorgrams, see Fig. S5).

We next investigated whether the B3H assay could be used to create designer BsAbs by direct selection of combinatorial libraries. Our objective was to select dual-antigen binders that interacted with a different leucine zipper, namely the leucine zipper domain of c-Jun (JunLZ)^15^, while retaining affinity towards gpD. We first replaced the gene encoding the Gcn4 leucine zipper in pTG-Fv_2_-gB with the gene encoding the JunLZ leucine zipper, yielding plasmid pTJ-Fv_2_-gB. Importantly, MC4100A cells transformed with this new plasmid were sensitive to Carb (Fig. 4A). Given that a band corresponding to the full-length gB chimera was lacking in the periplasm (Fig. 4B), we conclude that no complex between TJ, Fv2, and pG was formed, which would be required to export Bla to the periplasm. Taken together, these results indicate that the scFv-GCN4 domain in Fv_2_ was specific to Gcn4 and did not cross-react with JunLZ. Indeed, enzyme-linked immunosorbent assay (ELISA) confirmed that purified Fv_2_ bound strongly to immobilized Gcn4 but not JunLZ (Fig. 4C).

To reprogram the specificity of the Fv_2_ from Gcn4 to JunLZ, we randomized three important antigen-binding residues (GLF) in complementarity determining region 3 of the V_H_ domain (CDR-H3) of scFv-GCN4 using an NNK strategy (Fig. S4A). The scFv-GCN4 gene library was cloned in pTJ-Fv_2_-gB, resulting in the plasmid library pTJ-Fv_2_(NNK)-gB that was used to subsequently transform MC4100A cells. A total of ~8 × 10^3^ library clones were selected on plates containing 20 μg/mL Carb and thirty Carb-resistant clones were picked randomly for further analysis. The plasmids from these positive clones were isolated and used to retransform fresh cells to confirm the growth phenotype. Three of these clones had a clear growth advantage compared to MC4100A cells carrying pTJ-Fv_2_-gB (Fig. S4B). Sequencing of the CDR-H3 regions of these positive clones revealed three unique amino acid sequences, namely WQL, PAP and TFL (Fig. S4A). We focused on Fv_2_(WQL) because this clone conferred the strongest growth advantage to cells (Fig. 4A and Fig. S4B). Immunoblotting revealed that Fv_2_(WQL) but not the parental Fv_2_ was localized in the periplasm when JunLZ was co-expressed as one of the antigens (Fig. 4B). Consistent with the growth phenotypes, only cells expressing Fv_2_(WQL) accumulated full-length gB in the periplasm (Fig. 4B). It should be noted that a smaller ~28-kDa band appeared in the periplasm of control cells (Fig. 4B). We speculate that this band corresponds to a proteolytic product of the gB chimera that somehow becomes localized in the periplasm. This localization clearly does not involve the hitchhiker mechanism as the Fv_2_ proteins in these cases are localized exclusively in the cytoplasm. Hence, export of this unidentified fragment must proceed by an undetermined mechanism. The absence of all or most of Bla in this truncation product can be inferred from the fact that the cells producing this truncation product exhibit strong growth inhibition in the presence of Carb. Importantly, our results demonstrate that antigen-binding specificity of one of the scFv domains could be reprogrammed without compromising the antigen-binding activity of the other. Moreover, the growth advantage and hitchhiker export conferred by Fv_2_(WQL) was lost when the target antigen was changed to Gcn4 (Fig. 4A and B, respectively), indicating that the WQL substitution created a new CDR-H3 that was highly specific for JunLZ. In further support of this exquisite specificity, ELISA experiments revealed strong binding between purified Fv_2_(WQL) and JunLZ whereas no measurable binding above background was observed between Fv_2_(WQL) and Gcn4 (Fig. 4C).

The equilibrium dissociation constant, *K*_D_, for the interaction between Fv_2_(WQL) and JunLZ was 6.4 × 10^−7^ M as determined by Biacore analysis, which was measurably higher than that of the parental Fv_2_ for Gcn4 (*K*_D_ = 3.1 × 10^−8^ M) (Fig. 4D and Fig. S5). Nonetheless, isolation of a strong JunLZ binder after just a single round of highly focused mutagenesis (i.e., targeting of only 3 residues in CDR-H3) and selection reveals the potential of the B3H assay. Moreover, the *K*_D_ of Fv_2_(WQL) for gpD was 4.2 × 10^−5^ M, which compared favorably to the *K*_D_ values measured for the parental Fv_2_ or scFv-D10 against gpD (8.7 × 10^−6^ M and 2.2 × 10^−5^ M, respectively) (Fig. 4D and Fig. S5). Hence, reprogramming Fv_2_ to bind JunLZ was accomplished without significantly compromising gpD binding, thereby demonstrating the potential of the B3H assay for rapid isolation of proteins with dual affinity.

## Discussion

In this study, we uncovered a naturally occurring heterotrimer co-translocation process involving the aldehyde oxidoreductase PaoABC, which is exported by the Tat translocase as a ternary complex. Specifically, the PaoB and PaoC subunits, each lacking a discernable export signal, associate with the signal peptide-containing PaoA subunit to be translocated together across the cytoplasmic membrane. This unique mode of export whereby a polypeptide lacking a signal peptide can be effectively translocated in a piggyback fashion on another polypeptide containing a signal peptide is used by at least one other Tat substrate, namely the *E. coli* hydrogenase HybOC^9^, as well as by yeast and mammalian peroxisomes, which can translocate homo-oligomeric proteins (thiolase dimers and chloramphenicol acetyl transferase trimers) across their membranes^28^. In *E. coli*, at least five additional Tat substrates (out of the 27 proteins in *E. coli* known or predicted to bear N-terminal Tat signal peptides) may also be translocated as heterodimers: DmsAB, FdnGH, FdoGH, HyaAB, YnfEG, and YnfFG^10^. Some of these (HybO, HyaA, FdnH and FdoH) are components of larger multisubunit hydrogenase or formate dehydrogenase respiratory complexes that exhibit a heterotrimeric αβγ structure^29^. However, in these examples, only the αβ complex (e.g., FdnGH) is predicted to be exported as a preformed unit by a Tat signal peptide located on only one of the subunits; the γ-subunit (e.g., FdnI) is a signal recognition particle (SRP)-dependent integral membrane protein. Thus, to our knowledge, PaoABC is the only αβγ heterotrimer identified so far whose complete export depends on the Tat pathway. And while no upper limit has been determined for the size of a molecule that can be handled by the Tat translocase, PaoABC represents one of the largest structures (135-kDa) exported to date.

The translocation of PaoABC, like its HybOC counterpart, appears to involve an interesting QC phenomenon whereby absence of any one of the subunits prevents export of the others. The fact that the signal peptide itself is necessary but not sufficient for translocation suggests the involvement of an accessory factor, such as a dedicated molecular chaperone, that helps to coordinate PaoABC maturation (i.e., cofactor insertion, subunit assembly) and prevent wasteful export of misfolded or immature enzymes. Indeed, such dedicated ‘proofreading' chaperones have been identified for many Tat substrates, including αβ heterodimers whose export follows the hitchhiker mechanism^10^. In the case of HybOC, the HybE chaperone binds to the Tat signal peptide of HybO, probably acting to mask the signal from the Tat translocase while simultaneously preventing premature folding during cofactor loading^30^. Likewise, PaoD, is predicted to fulfill this role for PaoABC by participating in the modification and insertion of the Moco cofactor and coordinating these events with oligomer export^17^. In addition to dedicated chaperones, it is also plausible that the folding and assembly state of PaoABC may be monitored at a later stage in the export process. For example, the Tat translocase itself appears to have an inbuilt ability to discriminate between folded and mis/unfolded substrate proteins, allowing export of only the former^2^,^13^.

The interdependence of each subunit for export also implies direct contact between the subunits and favors a model in which the formation of a complex precedes translocation. In support of this model, we obtained several lines of evidence that PaoA, PaoB, and PaoC form a stable αβγ heterotrimer *in vivo*, in agreement with previous studies^17^,^18^. Interestingly, our BN-PAGE analysis revealed the formation of a possible (αβγ)_2_ dimer of PaoABC trimers, which could also be seen as a very minor product in the gel filtration profile. While further experiments are needed to unequivocally establish (αβγ)_2_ complex formation, such a complex is not entirely unexpected in light of other molybdoenzymes in the xanthine oxidase (XO) family, of which PaoABC is a member. For example, the xanthine dehydrogenase (XDH) system in *Rhodobacter capsulatus* exists as a non-covalent (αβ)_2_ dimer of heterodimers^31^, where the α subunit XdhA is homologous to the PaoAB complex and the β subunit XdhB is homologous to PaoC.

From a technological standpoint, a notable outcome of the studies described here was the creation of a B3H selection system for studying and engineering ternary protein complexes. We demonstrated that ternary protein interactions could be readily detected and optimized simply by selective plating of bacterial cells, a process that obviates the need for purification or immobilization of either binding target. In particular, we showed that the B3H selection was compatible with BsAbs (e.g., bispecific Fv_2_), which have great potential as biotherapeutics due to their dual target recognition enabling simultaneous inhibition of multiple cell surface receptors, cross-linking of two receptors, and recruitment of effector cells^32^,^33^,^34^. Because most BsAb formats, in particular tandem scFvs, are relatively small, aglycosylated proteins, their production is ideally suited for a simple, cost-effective host organism like *E. coli*. However, attempts to produce functional tandem scFvs in *E. coli* have been largely unsuccessful^35^,^36^,^37^,^38^, prompting a reliance upon eukaryotic host cells for improved functional expression^35^. Moreover, due to the lack of available techniques for selecting binders against two antigens simultaneously, BsAbs are typically isolated for their affinity to each antigen independently. This can be problematic because molecular recombination of two pre-existing, high-affinity binding proteins may yield a non-functional BsAb due to steric hindrance issues upon binding antigen or misfolding of the bispecific protein itself.

Notably, the B3H technology described here has the potential to overcome these challenges. First, simultaneous interaction between the dual-affinity BsAb and its two cognate antigens is required to confer selective growth. Second, the intrinsic folding QC of the Tat translocase^2^,^13^, which only exports properly folded, nonaggregating proteins, should favor the isolation of stable and soluble BsAbs. While not demonstrated here, we predict that our B3H selection system could be used to select for *trans-*acting factors (e.g., molecular chaperones) that promote folding, solubility and/or stability of BsAbs, such as *E. coli* FkpA^39^. Finally, in addition to BsAb development, we envision that the B3H technology could easily be leveraged for the creation of bifunctional adaptor proteins that (i) bring together two activities for rewiring cellular signaling or colocalizing metabolic pathways^40^,^41^, (ii) support orthogonal affinity purification strategies using two different matrices^42^, and (iii) function as immunoprobes in diagnostic assays^34^.

Finally, it should be pointed out that a small handful of three-hybrid assays in yeast and bacteria have been reported^43^,^44^,^45^,^46^,^47^. In every case, the assays rely on the reconstitution of a transcriptional activator that subsequently turns on expression of a reporter gene. This assay format has proven to be prone to a high frequency of false positives that arise from spurious transcriptional activation^48^, with >50% of the data generated using a transcription-based format likely to be false positives^49^. Because our assay does not involve DNA binding, it is not susceptible to spurious transcriptional activation. Another advantage of our B3H approach is that the Tat system only exports correctly folded, non-aggregated proteins and protein complexes^13^,^15^, thereby providing a built-in safeguard against false positives arising from the interaction of misfolded proteins. Also, because the assay requires the complex to remain associated during membrane transport, it stands to reason that relatively weak interactions will not be selected, which is especially advantageous for antibody development applications such as described here.

## Methods

### Bacterial strains and growth conditions

All strains used in this study are given in Supplementary Table S1. Parental strain *E. coli* BW25113^50^ and isogenic Keio derivatives JW0280 (BW25113 Δ*paoA*::Kan), JW0279 (BW25113 Δ*paoB*::Kan), and JW0278 (BW25113 Δ*paoC*::Kan)^51^ were used for PaoABC expression experiments. To eliminate the possibility of interference from endogenous *Pao proteins*, a triple knockout strain was prepared by sequential knockout of *paoB* and *paoC* in JW0280. This involved transformation of JW0280 with plasmid pCP20 to excise the Kan resistance gene in *paoA* as described^50^. The resulting strain was then subjected to P1 transduction using JW0279 (Δ*paoB*::Kan) as donor strain, followed by removal of the Kan marker in *paoB* using pCP20. Finally, the *paoC* gene was knocked out by P1 transduction using JW0278 as donor strain, resulting in strain HL0281 (BW25113 Δ*paoAB*-*paoC*::Kan). Wild-type *E. coli* MC4100 and its isogenic Δ*tatC* derivative called B1LK0^22^ were used for some PaoABC experiments. MC4100A (MC4100 *ara*^+^) and B1LK0A (B1LK0 *ara*^+^)^52^ were used for all BH3 experiments. *E. coli* JM109 was used for cloning genes while *E. coli* BL21(DE3) (Novagen) and SHuffle T7 Express (New England Biolabs) were used for cytoplasmic expression and purification of MBP-Gcn4, MBP-JunLZ, and all Fv_2_/scFv constructs. Typically, cultures were grown in Luria-Bertani (LB) medium supplemented with the appropriate antibiotic, and protein expression was induced when the cells reached OD_600_ ≈ 0.4–0.6 with isopropyl β-D-1-thiogalactopyranoside (IPTG; 0.1–0.5 mM) or arabinose (0.2% w/v) depending on the plasmid used. Antibiotics were provided at the following concentrations: chloramphenicol (Cm), 20 μg/mL; kanamycin (Kan), 50 μg/mL; ampicillin (Amp), 100 μg/mL; Carb, 0–1,000 μg/mL for selection.

### Plasmid construction

All plasmids used in this study are given in Supplementary Table S1.

To generate a synthetic *paoABC* operon, amplicon 1 (vector region-RBS-PaoA-c-Myc), amplicon 2 (c-myc-RBS-PaoB-FLAG), and amplicon 3 (FLAG-RBS-PaoC-HA-stop-vector region) were PCR amplified from *E. coli* genomic DNA using primers that appended overlapping regions (Fig. S1A). The resulting three PCR products and linearized pTrc99Y were joined *in vivo* by the yeast lazy-bone assembly method^53^, resulting in plasmid pPaoABC (Fig. S1B). Omission of each one of the *pao* genes in subsequent gene manipulations yielded plasmids pPao(ΔA)BC, pPaoA(ΔB)C, and pPaoAB(ΔC). The plasmid pPaoAhBC, encoding a 6xHis-tagged version of PaoA, was generated from pPaoABC by exchanging the DNA between XbaI/NotI encoding PaoA with a DNA fragment encoding PaoA-6xHis. To generate the B3H selection plasmid, amplicon B1 (vector region-RBS-ssTorA-Gcn4-c-Myc), amplicon B2 (c-Myc-RBS-scFv-GCN4-GS linker), amplicon B3 (GS linker-scFv-D10-FLAG-RBS-N-terminal gpD), amplicon B4 (N-terminal gpD-HA), and amplicon B5 (HA-mature Bla-vector region) were PCR amplified from laboratory stock plasmid DNA^15^,^27^ using primers that appended overlapping regions (Fig. S3A). The resulting five PCR products and linearized pBAD18-Cm were assembled using Gibson assembly (New England Biolabs) according to the manufacturer's instructions, resulting in plasmid pTG-Fv_2_-gB (Fig. 3B). Plasmid pTG-gB was constructed from pTG-Fv_2_-gB by omitting the tandem scFv portion in subsequent gene manipulations. Plasmid pT(KK)G-Fv_2_-gB was generated using a QuikChange site-directed mutagenesis kit (Agilent) with two antisense primers (forward: 5′-cga tct ctt tca ggc atc aaa gaa gcg ttt tct ggc aca act cgg cgg-3′; reverse: 5′-ccg ccg agt tgt gcc aga aaa cgc ttc ttt gat gcc tga aag aga tcg-3′). Plasmid pTJ-Fv_2_-gB was generated from pTG-Fv_2_-gB by first exchanging Gcn4 with JunLZ and then transferring the entire insert into pBAD18-Cm. For expression and purification of the target antigens Gcn4 and JunLZ, plasmid pET28-MBP-TEV was constructed by PCR amplifying the DNA encoding *E. coli* maltose-binding protein (MBP) with a C-terminal TEV cleavage site added by primer extension. The resulting PCR product was ligated between NdeI/BamHI sites of pET28a(+) (Novagen). Plasmids pET28-MBP-Gcn4 and pET28-MBP-JunLZ were generated by inserting PCR-amplified Gcn4 and JunLZ into BamHI/HindIII restriction sites of pET28-MBP-TEV. For expression and purification of gpD, plasmid pET28-gpD was constructed by double ligation of dimerizing primers encoding a 6xHis tag and HA tag with 5′ NcoI and 3′ NdeI overhangs and PCR-amplified gpD with 5′ NdeI and 3′ HindIII restriction sites into pET28a(+) between NcoI/HindIII restriction sites. For cytoplasmic expression and purification of Fv_2_ and scFv proteins, recombinant antibody genes were PCR amplified from laboratory stock plasmid DNA^15^,^27^ during which a C-terminal FLAG tag was introduced by using primer extension. The resulting PCR products were then ligated into pET28a(+) between NcoI/HindIII restriction sites without a stop codon in order to include the C-terminal 6xHis-tag in pET28a(+). This yielded pET28-scFv-GCN4, pET28-scFv-GCN4(WQL), pET28-scFv-D10, pET28-Fv_2_ and pET28-Fv_2_(WQL), where each included dual C-terminal FLAG/6xHis tags. All plasmids were confirmed by DNA sequencing.

### Library construction

Random mutagenesis using degenerate (NNK) forward primers and degenerate (MNN) reverse primers was used to create a 3-residue library containing mutations in the first three amino acids of the CDR-H3 (XXXDY) of the scFv-GCN4 domain of Fv_2_. Random forward primers were encoded by the sequence 5′-gat act gct ctc tat tac tgc gtg aca NNK NNK NNK gac tac tgg ggg cag ggc acg ctg gtt g-3′, where N is an equimolar mixture of all four nucleotides and K is an equimolar mixture of G and T, and random reverse primers were encoded by the sequence 5′- caa cca gcg tgc cct gcc ccc agt agt cMN NMN NMN Ntg tca cgc agt aat aga gag cag tat c-3′, where M is an equimolar mixture of A and C and N is an equimolar mixture of all four nucleotides. These primers were used to generate a mutant library from plasmid pTG-Fv_2_-gB as template according to the QuikChange mutagenesis protocol (Agilent). After 18 cycles of amplification, randomized scFv-GCN4(XXX) sequences were amplified with forward primer (5′- ggt cgc tag cat gcg aga tat cgt tat gac c -3′) and reverse primer (5′-tca agg tcg acc gat ccg cca cc-3′). The resulting PCR products, encoding a DNA library of scFv-GCN4(XXX) sequences, were cloned into pTJ-Fv_2_-gB using NheI and SalI restriction enzymes. The resulting plasmid was used to transform *E. coli* MC4100 and then selected on LB agar containing Cm to recover clones containing library plasmids. Library cells were pooled and their plasmids were isolated for selection experiments.

### Selective growth assays and library selection

For B3H experiments, cells carrying pTG-Fv_2_-gB plasmid or equivalent controls were grown overnight in LB containing 20 μg/mL Cm. Screening of cells was performed by spreading an equivalent number of serially diluted overnight cells directly onto LB agar plates supplemented with 0.2% (w/v) arabinose and 0, 20 or 50 μg/mL Carb and incubating at 30°C for 24 h. Library selections were performed by electroporating the tricistronic library plasmid pTJ-Fv_2_(NNK)-gB into *E. coli* MC4100 cells followed by direct plating on LB agar supplemented with 0.2% (w/v) arabinose and 10 μg/mL Carb. Thirty randomly chosen Carb-resistant clones were subjected to further characterization by spreading an equivalent number of serially diluted overnight cells directly onto LB agar plates supplemented with 0.2% (w/v) arabinose and 10 μg/mL Carb and incubating at 30°C for 48 h. Additionally, these thirty clones were subjected to sequencing to eliminate false positives from direct recombination of the TorA-derived signal peptide with Bla. Finally, selected cells were confirmed by spot plating 5 μL of overnight cells that had been normalized in fresh LB to OD_600_ ≈ 1 onto LB agar plates supplemented with 0.2% (w/v) arabinose and 0–50 μg/mL Carb and incubating at 30°C for 48 h.

### Subcellular fractionation and Western blot analysis

The subcellular fractionation was performed as described elsewhere^54^. Briefly, to prepare subcellular fractions for Western blot analysis, we pelleted and washed 15 mL of induced culture with subcellular fractionation buffer (30 mM Tris–HCl, 1 mM EDTA, and 0.6 M sucrose). Cells were resuspended in 1 mL subcellular fractionation buffer and then incubated for 10 min at room temperature and spun down. After addition of 266 μL of 5 mM MgSO_4_, cells were incubated for 10 min on ice. Cells were spun down, and the supernatant was taken as the periplasmic fraction. The pellet was treated with BugBuster Master Mix (Novagen) for 20 min at room temperature. Following centrifugation at 12,000 rpm at room temperature for 5 min, the second supernatant was taken as the soluble cytoplasmic fraction, and the pellet was retained as the insoluble fraction. Soluble lysates were normalized using a total protein assay (Bio-Rad) with BSA as standard and samples were normalized to load 20 μg total protein per lane. Cytoplasmic and periplasmic proteins were separated by SDS-PAGE and Western blotted as previously described^13^. The following primary antibodies were used: rabbit anti-c-Myc clone 9E10 (1:5,000; Sigma); rabbit anti-HA (1:1500; Abcam); rabbit anti-GroEL (1:20,000; Sigma). Secondary anti-rabbit polyclonal antibodies conjugated to horseradish peroxidase (HRP) (1:2500; Promega) were used for detection. Also, mouse anti-FLAG-HRP conjugated to HRP (1:3,000; Sigma) and rabbit anti-His antibodies conjugated to HRP (1:10,000; Abcam) were used depending on affinity tags used.

### Protein purification

Gcn4 and JunLZ were expressed as MBP fusions from pET28-MBP-Gcn4 and pET-MBP-JunLZ, respectively, in the cytoplasm of *E. coli* BL21(DE3) cells. The resulting MBP fusions were each purified using amylose affinity chromatography. Briefly, soluble lysates were applied to amylose columns, and the columns were washed with five times the bead volume with a buffer containing 1 mM EDTA, 200 mM NaCl, 20 mM Tris/Cl (pH 7.4). Fusion proteins were eluted using a buffer containing 1 mM EDTA, 200 mM NaCl, 10 mM maltose, 20 mM Tris/Cl (pH 7.4). After purification, eluted MBP fusions were further desalted using a Zeba™ Spin Desalting Column (Fisher) into 20 mM Tris/Cl(pH.4). The gpD and scFv-D10 proteins were expressed from pET28-gpD and pET28-scFv-D10, respectively, in BL21(DE3) cells, while scFv-GCN4 and scFv-GCN4(WQL) were expressed in the cytoplasm of SHuffle T7 Express cells (New England Biolabs) from pET28-scFv-GCN4 and pET28-GCN4(WQL), respectively. All of these 6xHis-tagged proteins were purified using Ni-NTA HisTrap FPLC columns (GE Healthcare, ÄKTA) according to manufacturer's protocols. Briefly, soluble lysates were applied to Ni-NTA columns, and the columns were washed with five times the column volume with a buffer containing 50 mM NaH_2_PO_4_, 300 mM sodium chloride, and 20 mM imidazole (pH 8.0). The proteins were all eluted using a buffer containing 50 mM NaH_2_PO_4_, 300 mM sodium chloride, and 250 mM imidazole (pH 8.0). Following FPLC purification, eluted proteins were further desalted as described above. The bispecific proteins Fv_2_ and Fv_2_(WQL) were expressed in the cytoplasm of SHuffle T7 Express cells from pET28- Fv_2_ and pET28-Fv_2_(WQL), respectively. After cell lysis with Bugbuster™ (Novagen), cell debris was partially removed by centrifugation at 6000 × g. The resulting turbid supernatant was separated into soluble and insoluble fractions by centrifugation at 30,000 × g. To improve the recovery yield of the bispecifics without affecting folding, we resolubilized the insoluble fraction with 0.5% sarkosyl^55^. Resolubilized material was then mixed with the soluble fraction in HisTrap binding buffer to a final concentration of 0.1% sarkosyl. Also, Triton X-100 and CHAPS was added to these samples to a final concentration of 1% and 10 mM, respectively, to improve protein binding to the Ni-NTA resin^55^. The resulting soluble 6xHis-tagged Fv_2_ and Fv_2_(WQL) proteins were purified and desalted as described above. Final purity of all proteins was confirmed by SDS-PAGE (Fig. S6).

### Biochemical analysis of PaoABC complex formation

PaoA with a 6xHis-tag was co-expressed with PaoB-FLAG and PaoC-HA from plasmid pPaoAhBC and soluble lysates were prepared as described in the Materials and Methods. The 6xHis-tagged PaoA and associated proteins was purified using Ni-NTA protein purification spin columns (Qiagen) according to manufacturer's protocols. Briefly, soluble lysates were applied to the columns, and the columns were washed four times with a buffer containing 50 mM NaH_2_PO_4_, 300 mM sodium chloride, and 20 mM imidazole (pH 8.0). Associated proteins were eluted using a buffer containing 50 mM NaH_2_PO_4_, 300 mM sodium chloride, and 500 mM imidazole (pH 8.0). After spin-column purification, eluted samples were further desalted using a Zeba™ Spin Desalting Column (Thermo Scientific) into 20 mM Tris/Cl buffer (pH 7.4). Purified samples were then analyzed by SDS-PAGE and immunoblotting. Separation by BN-PAGE (Novex NativePAGE, Life Technologies) was performed as previously described^56^ and according to the manufacturer's protocols. Molecular weights on BN-PAGE were estimated by RF value using a native marker as standard.

### Enzyme-linked immunosorbent assay

Enzyme-linked immunosorbent assay (ELISA) was used to evaluate the binding of purified Fv_2_ proteins to Gcn4 and JunLZ. ELISA plates were coated overnight at 4°C with 50 μL/well of each antigen in PBS (10 μg/mL). Plates were then blocked at room temperature for 2 h with 2% nonfat milk in PBS. After washing plates with PBS supplemented with 0.1% Tween 20 (PBST), purified protein samples serially diluted in PBS with 50 μg/mL BSA (PBS-BSA) were added to the plates (50 μL/well). Plates were incubated for 1 h at room temperature and then washed with PBST. HRP-conjugated anti-His antibody (1:10,000; Abcam) in PBS-BSA was added to the plates (50 μL/well). After 1 h of incubation at room temperature, plates were washed and then incubated with SigmaFast OPD HRP substrate (Sigma) for 20 min. The reaction was quenched with 3N H_2_SO_4_, and the absorbance of the wells was measured at 490 nm.

### Surface plasmon resonance

Surface plasmon resonance (SPR) experiments were performed using a Biacore 3000 biosensor (GE Healthcare). Purified MBP-Gcn4, MBP-JunLZ and gpD were immobilized on CM5 sensor chips at 10 μg/ml using an amine coupling kit at pH 4.0 until reaching 1200 RU, 1000 RU, and 260 RU, respectively, according to the manufacturer's instructions. The binding analyses were carried out in HBS-EP Buffer (GE Healthcare Life Sciences) at a flow rate of 30 μl/min. Each Fv_2_ or scFv protein was associated with ligands for 3 min, and then dissociation phases were observed. During SPR experiments, flow cells were regenerated by injection of 10 mM glycine (pH 2.0) followed by thorough washing with the running buffer. Additionally, a blank cell on the same sensor chip was used as a reference to correct for non-specific binding. Kinetic parameters were calculated using BIAevaluation software 3.2. All sensorgrams were fitted to a 1:1 Langmuir binding model using a simultaneous non-linear program.

## Author Contributions

H.-C.L. and A.D.P. designed research, performed research, analyzed data, and wrote the paper. M.A.R. designed research, performed research, analyzed data. M.P.D. conceptualized project, designed research, analyzed data, and wrote the paper. All authors reviewed the manuscript.

## Supplementary Material

Supplementary Information Supplementary Information
