# Supplementary material for: An engineered genetic selection for ternary protein complexes inspired by a natural three-component hitchhiker mechanism
Source: Sci Rep. 2014 Dec 22;4:7570. doi: 10.1038/srep07570 (PMC4273604; doi:10.1038/srep07570)
Supplement: Supplementary Information [file srep07570-s1.pdf]

Supplementary Information File

"An engineered genetic selection for ternary protein complexes inspired by a natural three-component hitchhiker mechanism"

Hyeon-Cheol Lee, Alyse D. Portnoff, Mark A. Rocco and Matthew P. DeLisa

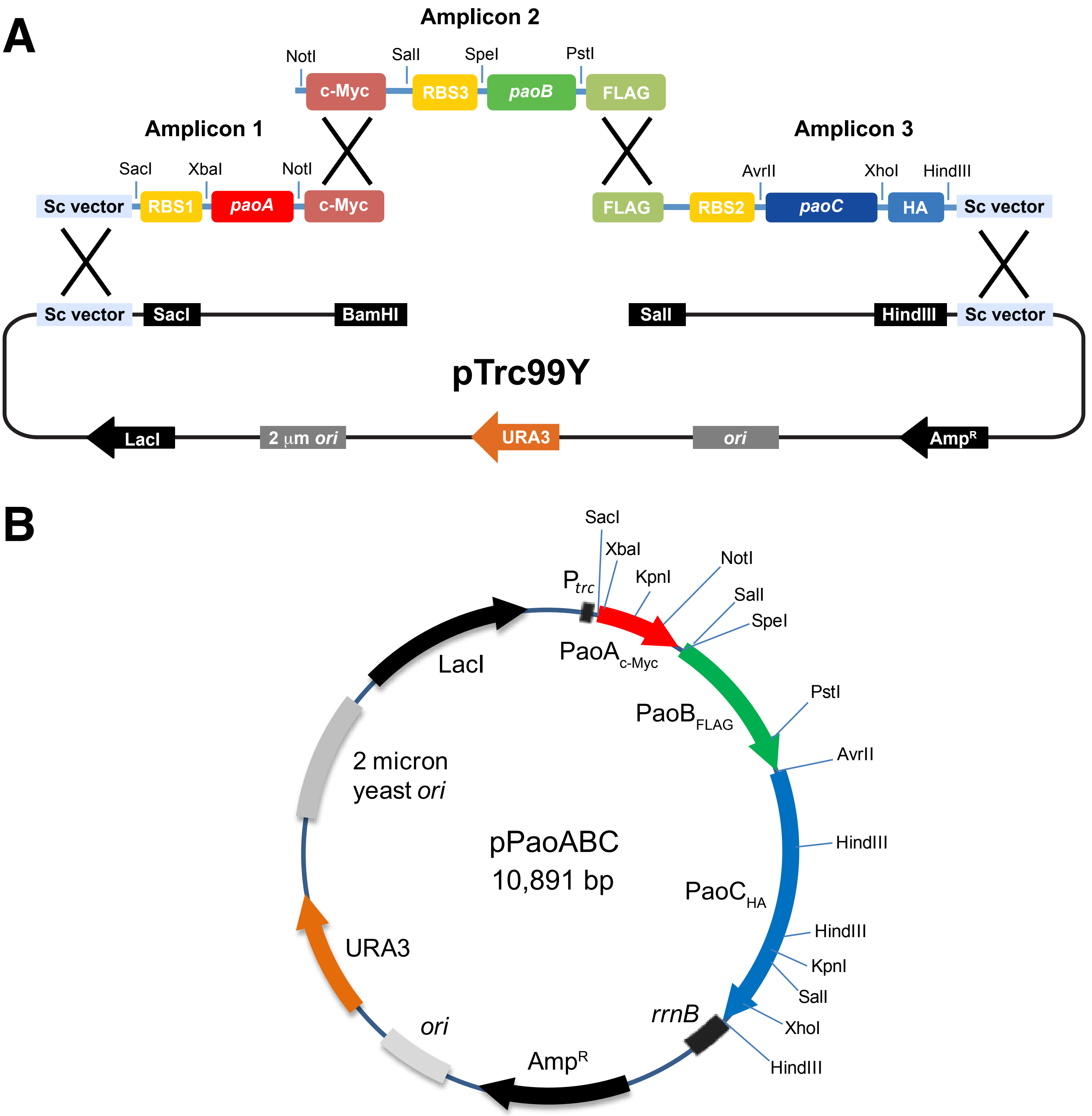

Supplementary Figure S1. Construction of synthetic *paoABC* operon. (A) Plasmid pPaoABC, encoding a synthetic operon comprised of the *paoABC* genes, was constructed using a yeast recombination cloning method. To generate pPaoABC, amplicon 1 (vector region-RBS-PaoA-c-Myc), amplicon 2 (c-myc-RBS-PaoB-FLAG), and amplicon 3 (FLAG-RBS-PaoC-HA-stop-vector region) were PCR amplified from *E. coli* genomic DNA using primers that appended overlapping regions. The resulting three PCR products and linearized pTrc99Y were joined *in vivo* by the yeast lazy-bone assembly method. (B) Plasmid map of pPaoABC.

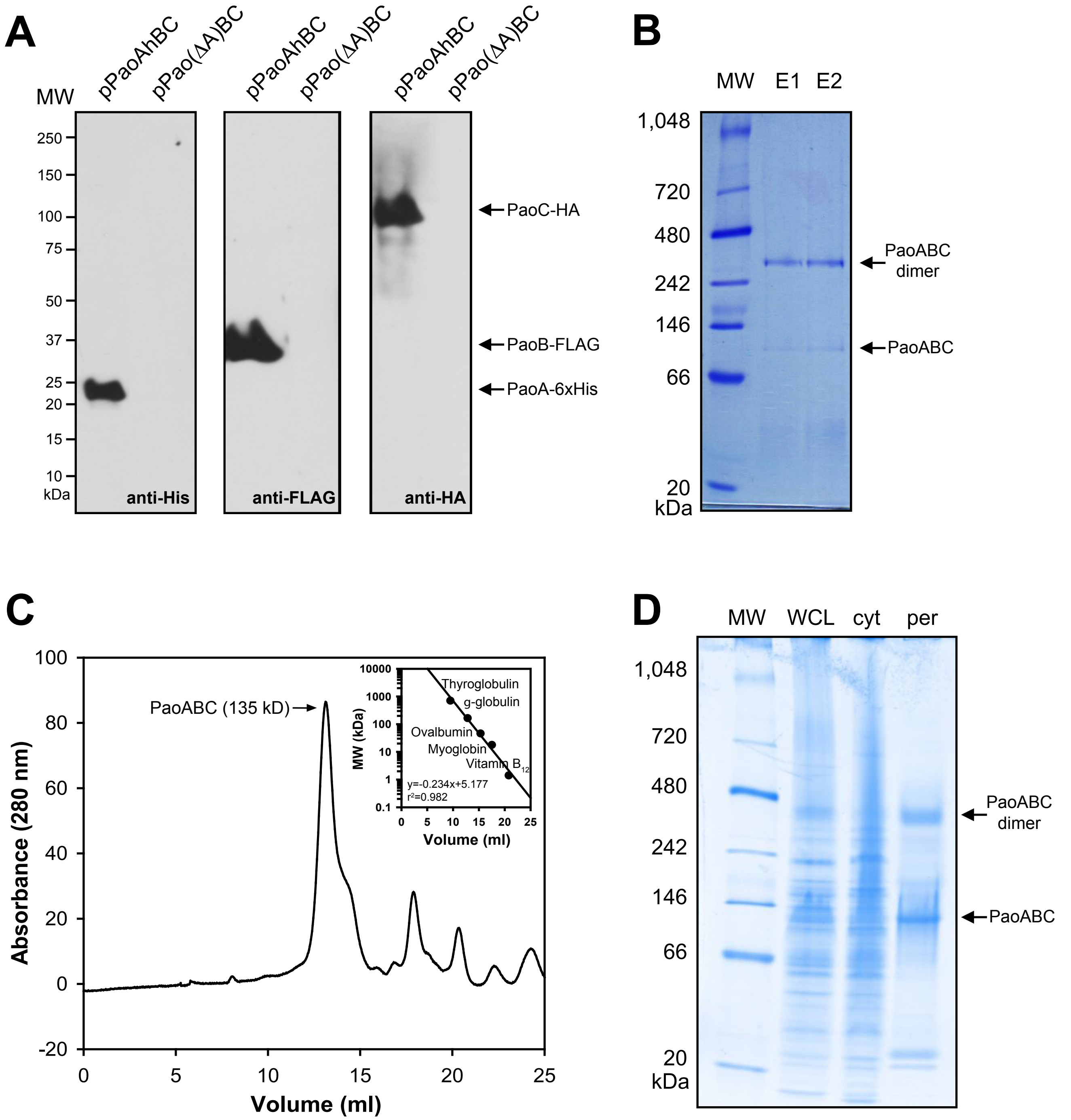

Supplementary Figure S2. Heteroassembly of PaoABC complex. (A) Western blot analysis of samples co-eluted with 6xHis-tagged PaoA. PaoB-FLAG and PaoC-HA were co-expressed with and without the 6xHis-tagged version of PaoA from plasmid pPaoAhBC in Δ*paoAB-paoC*::Kan cells. Ni-NTA elution fractions were probed with anti-His, anti-FLAG, and anti-HA antibodies to detect each individual Pao protein. Molecular weight (MW) markers are indicated on the left. (B) BN-PAGE of samples purified from soluble lysate derived from Δ*paoAB-paoC*::Kan cells expressing pPaoAhBC containing the 6xHis-tagged PaoA. MW markers are shown on the left. (C) Size exclusion chromatography of the periplasmic fraction prepared from Δ*paoAB-paoC*::Kan cells expressing pPaoABC. One hundred microliters of the periplasmic fraction was analyzed by size exclusion chromatography in 50 mM Tris and 200 mM NaCl (pH 7.5) using a Superdex 200 column HR 10/30 (Amersham Biosciences). Inset: plot of the standard proteins. Size exclusion chromatography markers (Bio-Rad): thyroglobulin (670 kDa) γ-globulin (158 kDa), ovalbumin (44 kDa), myoglobin (17 kDa), and vitamin B12 (1.35 kDa). (D) BN-PAGE of whole cell lysate (WCL), cytoplasmic (cyt) and periplasmic (per) fractions prepared from Δ*paoAB-paoC*::Kan cells expressing pPaoABC. MW markers are shown on the left.

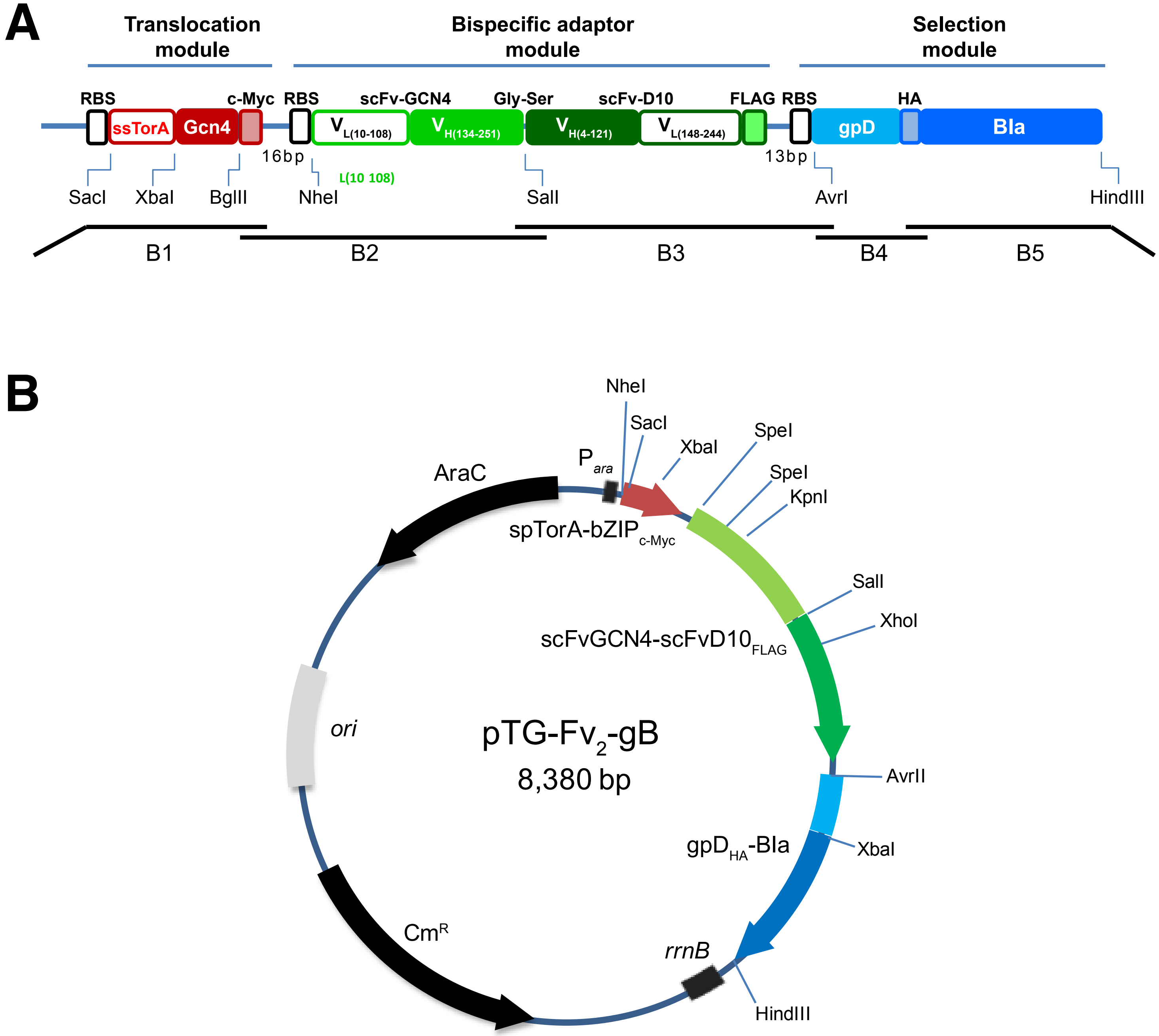

Supplementary Figure S3. Construction of B3H selection system plasmid. (A) Plasmid pTG-Fv2-gB was constructed using Gibson assembly. To generate pTG-Fv2-gB, amplicons B1 (vector region-RBS-ssTorA-Gcn4-c-Myc), B2 (c-Myc-RBS-scFv-GCN4-GS linker), B3 (GS linker-scFv-D10-FLAG-RBS-N-terminal gpD), B4 (N-terminal gpD-HA), and B5 (HA-mature Bla-vector region) were PCR amplified from laboratory stock plasmid DNA using primers that appended overlapping regions. The resulting five PCR products and linearized pBAD18-Cm were combined using Gibson assembly. (B) Plasmid map pTG-Fv_2-gB.

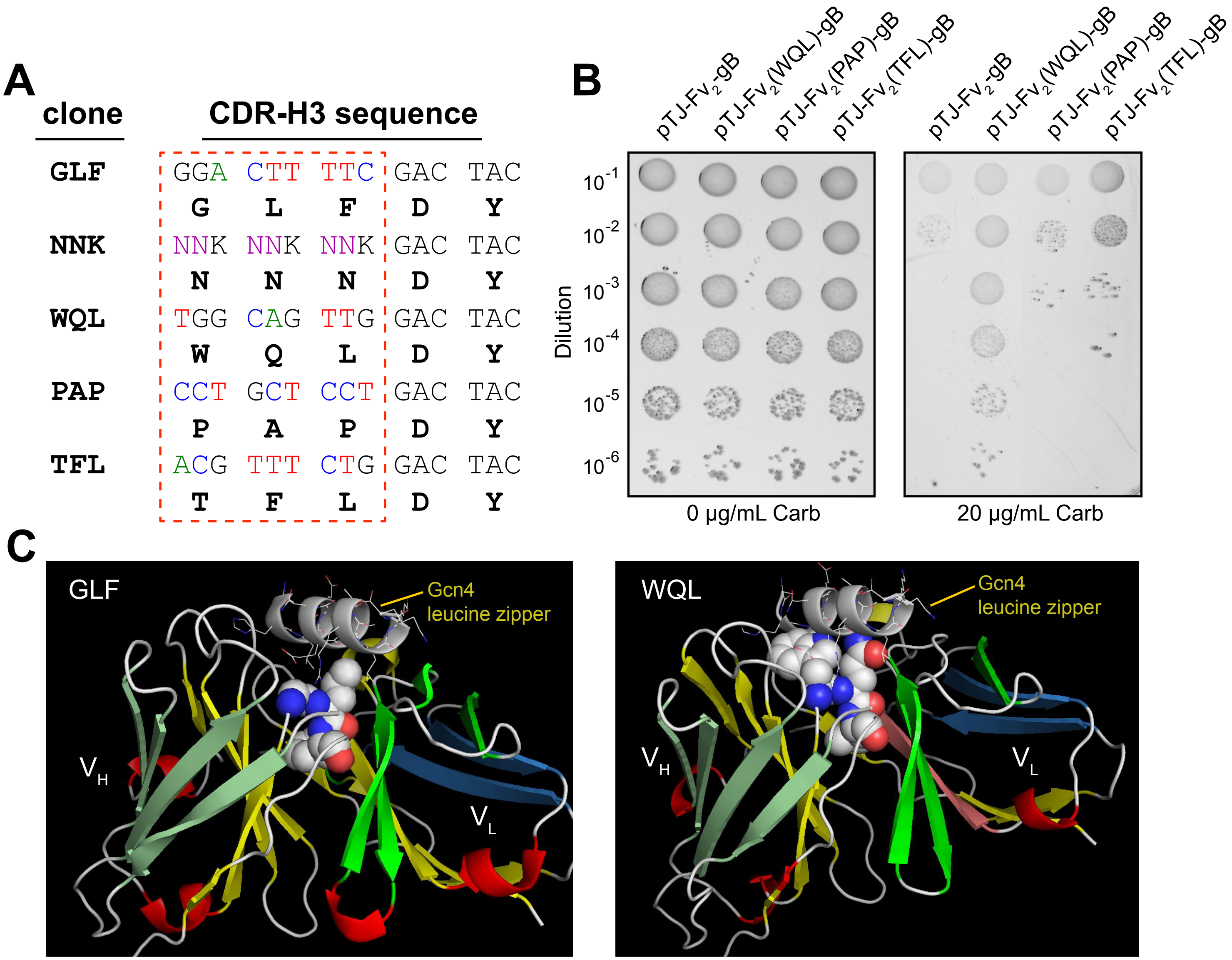

Supplementary Figure S4. Reprogramming antigen specificity using BH3-mediated genetic selection. (A) Three important antigen-binding residues (GLF) in the complementarity determining region 3 of the V_H domain (CDR-H3) were randomized for library generation. Sequences of positive clones selected from the NNK library in Fv_2. (B) Selective spot plating of serially diluted MC4100A cells co-expressing the parental Fv_2 or one of the three positive clones along with the JunLZ antigen from pTJ-Fv_2-gB. Overnight cultures were serially diluted in liquid LB and spot plated on LB-agar supplemented with Carb (0-20 µg/mL) and arabinose (0.2% w/v). (C) Comparison of the antigen-binding domain of GLF with that of the WQL clone. Models were generated in PyMOL™ from the original co-crystal structure of scFv-GCN4 with the Gcn4 leucine zipper. Steric clash with the Gcn4 antigen was observed when the WQL mutation was modeled place of GLF in the CDR-H3 region of the scFv-GCN4 structure.

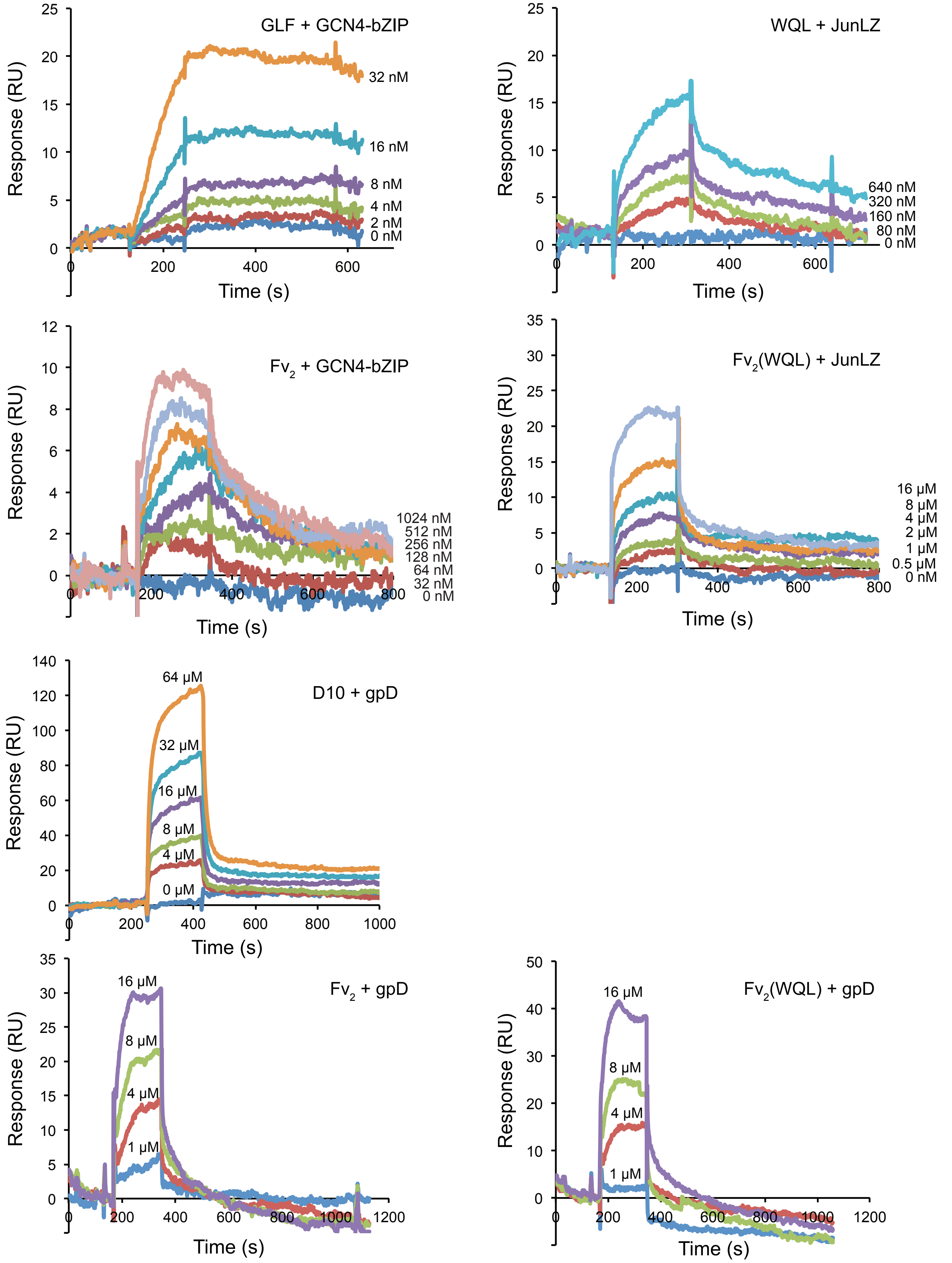

Supplementary Figure S5. Binding kinetics of recombinant antibodies against target antigens. Biacore sensorgrams generated for purified bispecific Fv_2s (wt Fv_2 and Fv_2(WQL) clone) or unfused scFvs (scFv-GCN4, scFv-GCN4(WQL) and scFv-D10) measured by SPR. The binding kinetics of bispecific Fv_2s or unfused scFvs were monitored using Biacore. Cognate antigens MBP-Gcn4, MBP-JunLZ and gpD were immobilized on CM5 chips and the response of varied concentrations of bispecific Fv_2s or unfused scFvs, given in each curve, was compared with an empty flow cell. Purity of all recombinant proteins was assessed by SDS-PAGE (see *SI* Fig. S6). Affinity values were obtained by fitting the equilibrium binding responses with a 1:1 Langmuir binding model using a simultaneous non-linear program. Representative results are depicted.

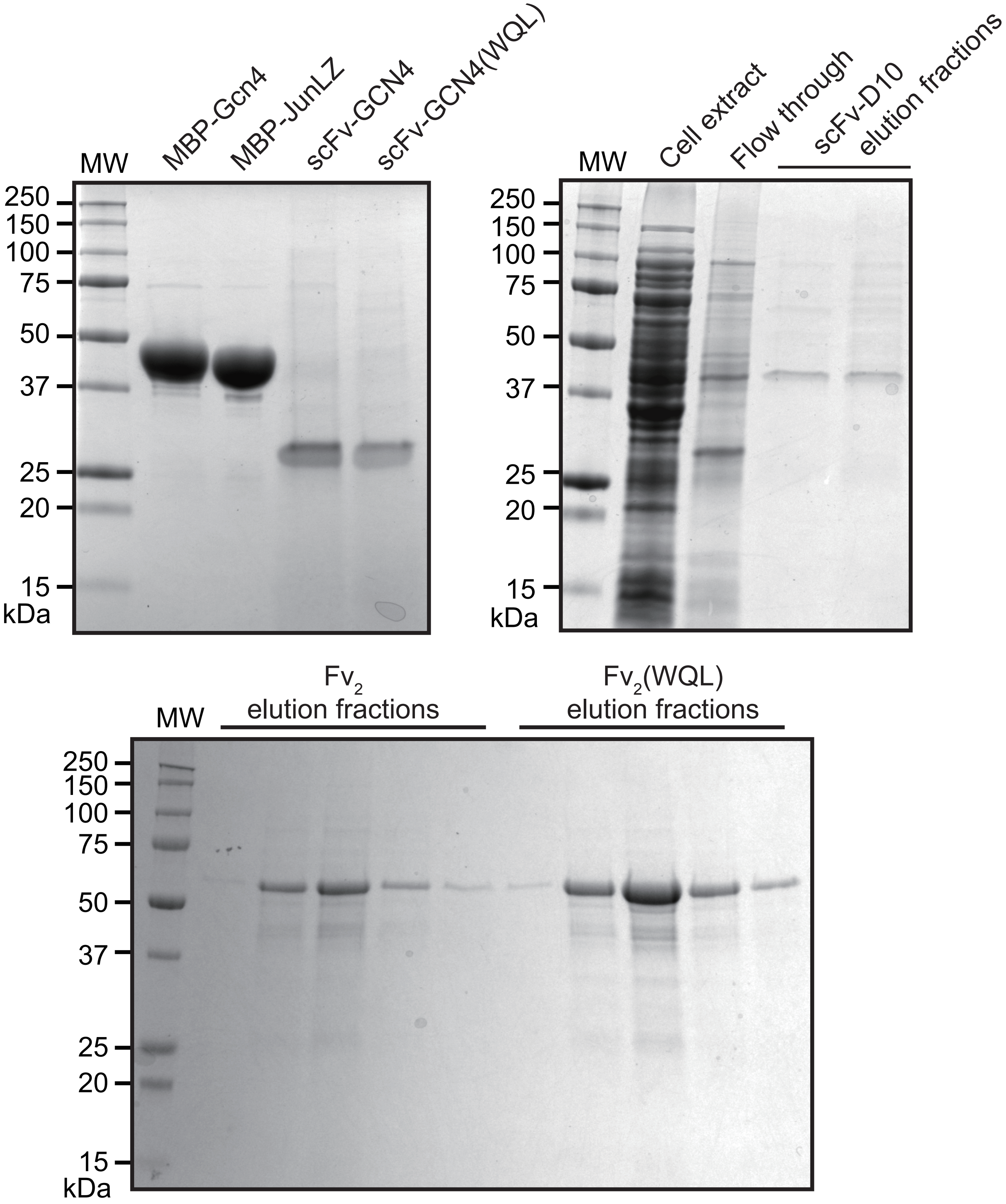

Supplementary Figure S6. Purification of recombinant antibodies and corresponding antigens. SDS-PAGE analysis of protein purity by Coomassie staining of recombinant antibodies and corresponding antigens to be used in Biacore analysis. Antigens MBP-GCN4 and MBP-JunLZ were purified using amylose affinity chromatography. All other proteins were purified using Ni-NTA affinity chromatography. Molecular weight (MW) markers are shown on left.

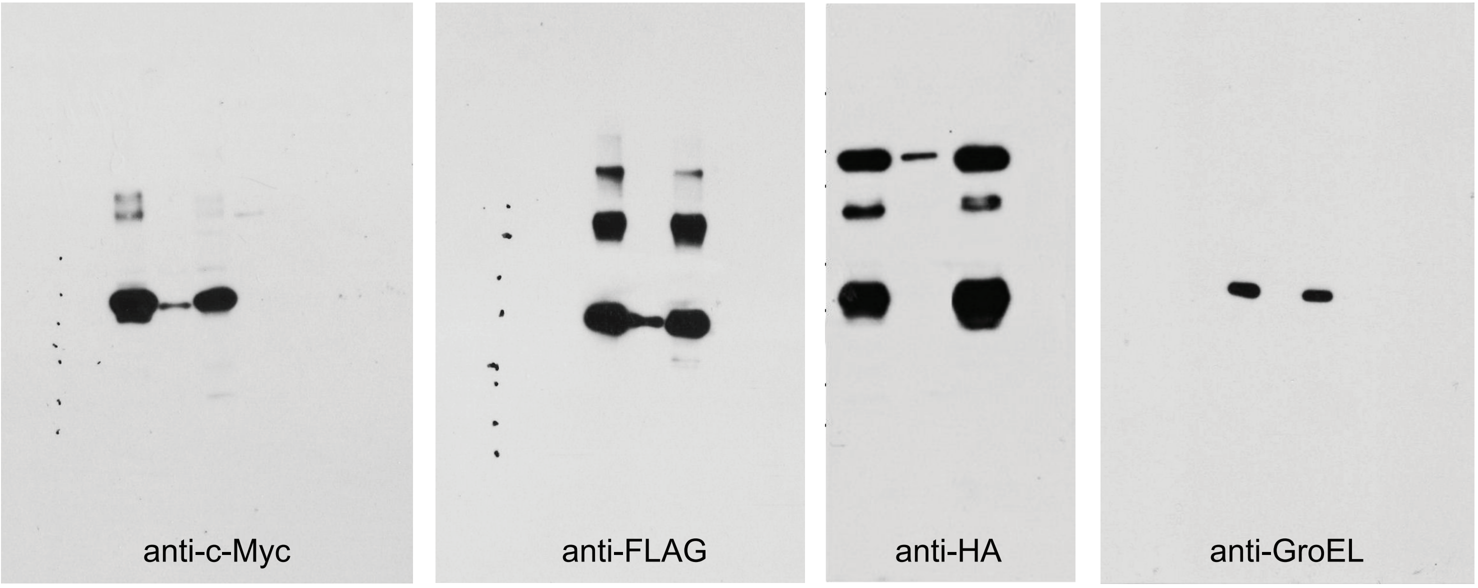

Supplementary Figure S7. Uncropped images corresponding to Figure 1.

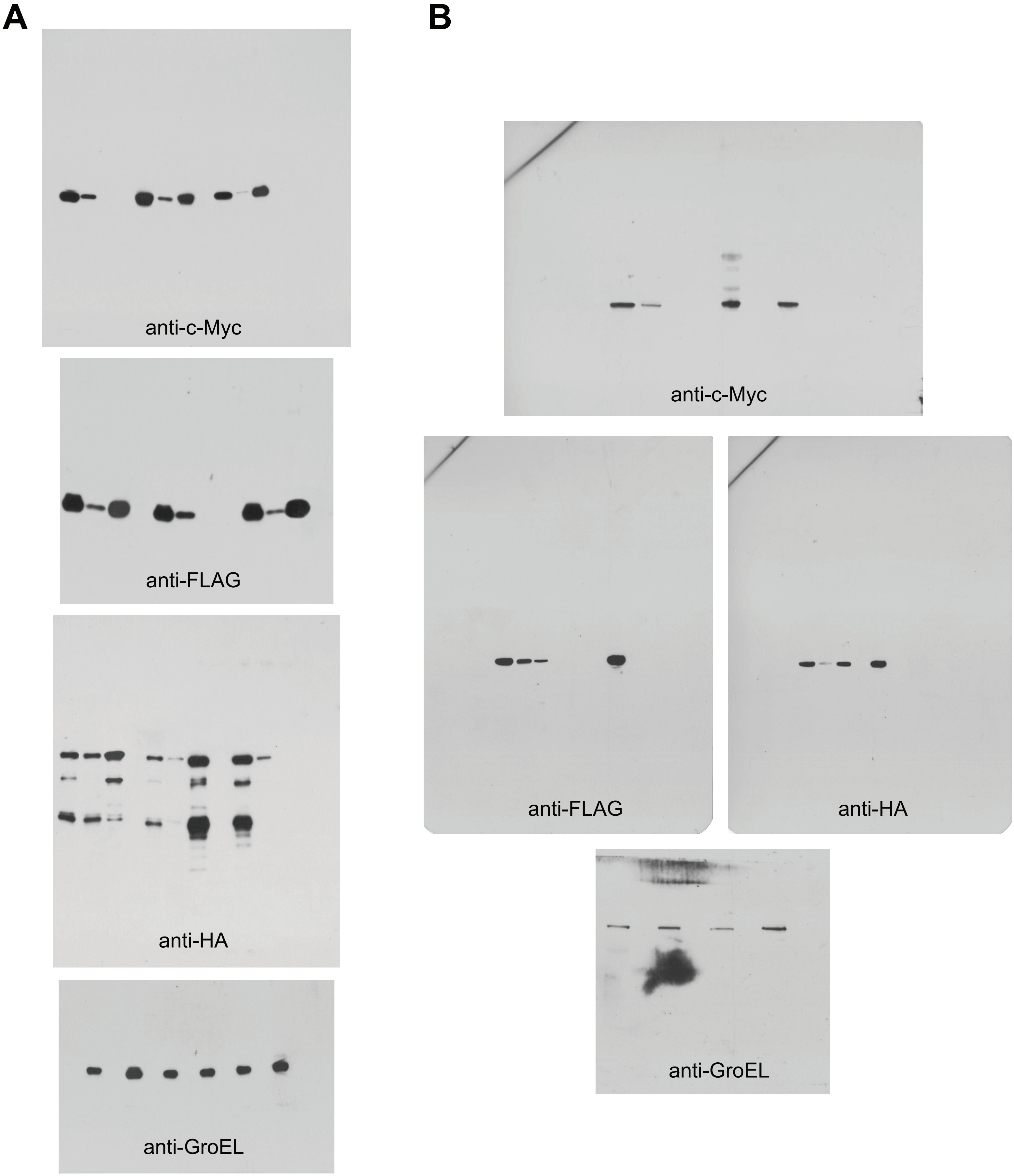

Supplementary Figure S8. Uncropped images corresponding to Figure 2.

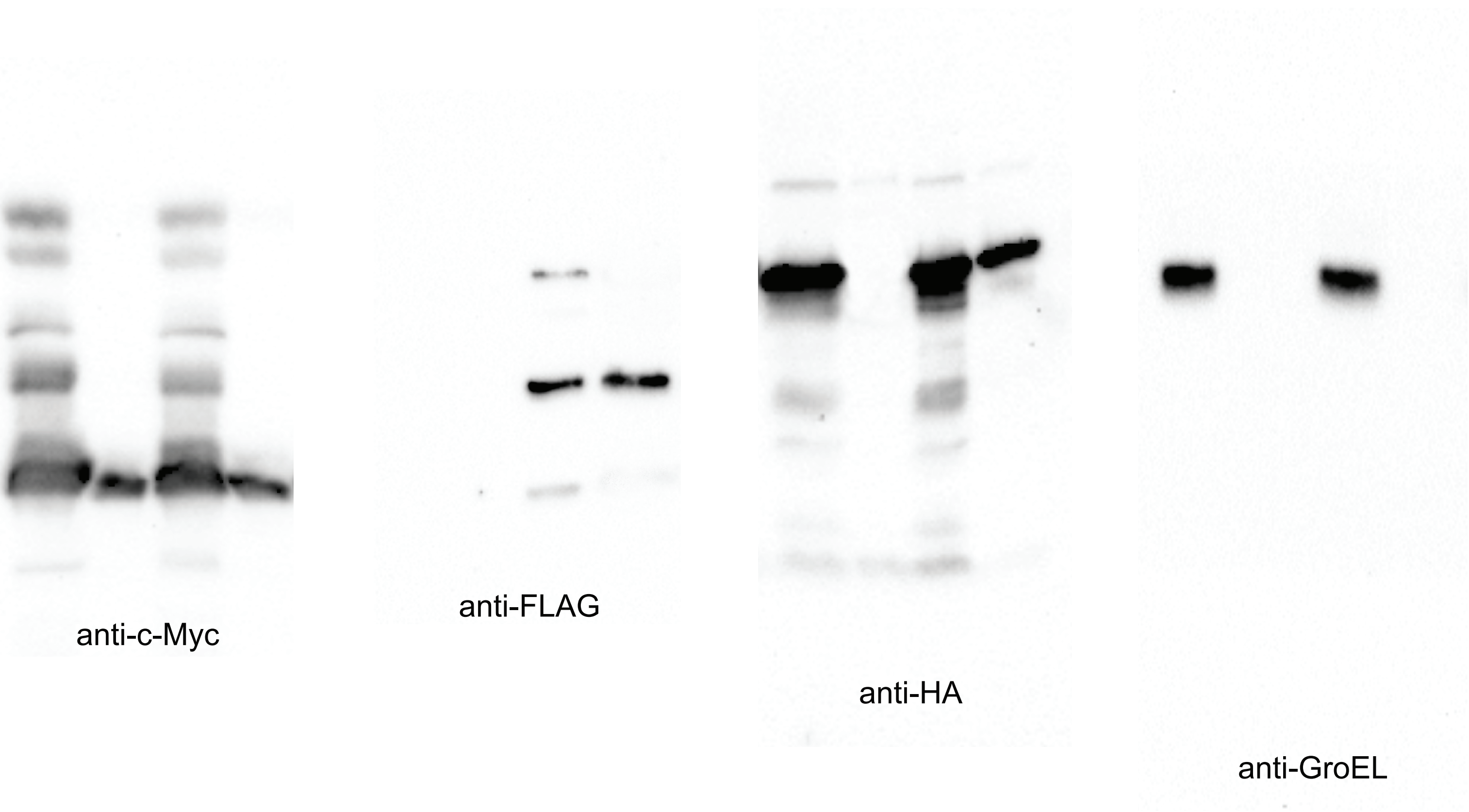

Supplementary Figure S9. Uncropped images corresponding to Figure 3.

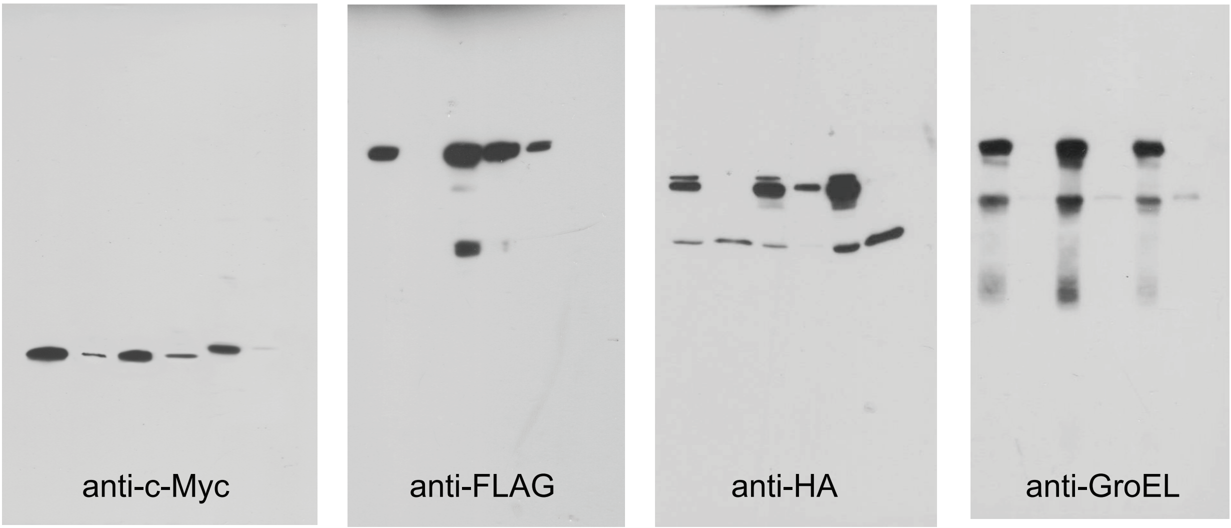

Supplementary Figure S10. Uncropped images corresponding to Figure 4.

Supplementary Table S1. Strains and plasmids used in this study

Strain or plasmid	Description	Source or reference
Strains		
BW25113	*lacI*^{q} *rrnB*$_{T14}$ Δ*lacZ*$_{WJ16}$ *hsdR514* Δ*araBAD*$_{AH33}$ Δ*rhaBAD*$_{LD78}$	(1)
JW0280	BW25113 Δ*paoA*::Kan	(2)
JW0279	BW25113 Δ*paoB*::Kan	(2)
JW0278	BW25113 Δ*paoC*::Kan	(2)
HL0281	JW0280 Δ*paoAB-paoC*::Kan	This study
MC4100	F′ *araD139* Δ(*argF-lac*)U169 *rpsL150* (Str^{r}) *relA1 flbB5301 deoC1 ptsF25 rbsR*	Laboratory stock
MC4100A	MC4100 *ara*$^{+}$	Laboratory stock
B1LK0	MC4100 Δ*tatC*	(3)
B1LK0A	B1LK0 *ara*$^{+}$	Laboratory stock
JM109	*endA1 glnV44 thi-1 relA1 gyrA96 recA1 mcrB*$^{+}$ Δ(*lac-proAB*) e14- [F' *traD36 proAB*$^{+}$ *lacI*^{q} *lacZ*ΔM15] *hsdR17*($r_K^- m_K^+$)	Laboratory stock
BL21(DE3)	F^{-} *ompT hsdSB*(rB^{-}, mB^{-}) *gal dcm* (DE3)	Novagen
Shuffle T7 Express	*fhuA2 lacZ::T7 gene1* [lon] *ompT ahpC gal λatt*::pNEB3-r1-*cDsbC* (Spec^{R}, *lacI*^{q}) Δ*trxB sulA11 R(mcr-73::miniTn10*—Tet^{S})2 [dcm] *R(zgb-210::Tn10*—Tet^{S}) *endA1* Δ*gor* Δ*(mcrC-mrr)114::IS10*	New England Biolabs
Plasmids		
pTrc99Y	P$_{trc}$ promoter; ColE1 *ori*, Amp^{r}; 2μm *ori*, ura3	Laboratory stock
pBAD18-Cm	P$_{BAD}$ promoter; pBR322 *ori*, Cm^{r}	(4)
pET28a(+)	P$_{T7}$ promoter; pBR322 *ori*, Kan^{r}	Novagen
pCP20	*FLP*$^{+}$, λ *c*i857^{+}, λ p_R Rep^{ts}, Amp^{R}, Cm^{R}	(5)
pPaoABC	Tricistronic expression of PaoA with C-terminal c-Myc tag, PaoB with C-terminal FLAG tag, and PaoC with C-terminal HA tag in pTrc99Y	This study
pPaoAhBC	Tricistronic expression of PaoA with C-terminal 6xHis tag, PaoB with C-terminal FLAG tag, and PaoC with C-terminal HA tag in pTrc99Y	This study
pPao(ΔA)BC	Bicistronic expression of PaoB with C-terminal FLAG tag and PaoC with C-terminal HA tag in pTrc99Y	This study
pPaoA(ΔB)C	Bicistronic expression of PaoA with C-terminal c-Myc tag and PaoC with C-terminal HA tag in pTrc99Y	This study
pPaoAB(ΔC)	Bicistronic expression of PaoA with C-terminal c-Myc tag and PaoB with C-terminal FLAG tag in pTrc99Y	This study
pTG-gB	Bicistronic expression of Gcn4 with N-terminal TorA signal peptide and C-terminal c-Myc tag, and gpD fused to the mature Bla in pBAD18-Cm	This study
pTG-Fv$_2$-gB	Tricistronic expression of ssTorA-Gcn4 with C-terminal c-Myc tag, Fv$_2$ (scFv-Gcn4 fused to scFv-D10) with C-terminal FLAG tag, and gpD-Bla with C-terminal HA tag in pBAD18-Cm	This study
pT(KK)G-Fv$_2$-gB	pTG-Fv$_2$-gB but with RR to KK substitution in TorA signal peptide	This study
pTJ-Fv$_2$(NNK)-gB	pTJ-Fv$_2$-gB but with randomized NNK in place of GLF in CDR-H3 in scFv-GCN4 sequence	This study
pTJ-Fv$_2$(WQL)-gB	pTJ-Fv$_2$-gB but with GLF to WQL substitution in CDR-H3 in scFv-GCN4 sequence	This study
pET28-MBP-GCN4	MBP-TEV fused to Gcn4 leucine zipper domain in pET28a(+)	This study
pET28-MBP-JunLZ	MBP-TEV fused to c-Jun leucine zipper domain in pET28a(+)	This study
pET28-gpD	gpD with N-terminal 6xHis in pET28a(+)	This study

pET28-scFv-GCN4	scFv-GCN4 cloned with C-terminal FLAG-6xHis in pET28a(+)	This study
pET28-scFv-GCN4(WQL)	scFv-GCN4(WQL) cloned with C-terminal FLAG-6xHis in pET28a(+)	This study
pET28-scFv-D10	scFv-D10 cloned with C-terminal FLAG-6xHis in pET28a(+)	This study
pET28-Fv_2	Fv_2 created by fusion of scFv-GCN4 to scFv-D10 with $(Gly_4Ser)_5$ linker and C-terminal FLAG-6xHis in pET28a(+)	This study
pET28-Fv_2(WQL)	Fv_2(WQL) created by fusion of scFv-GCN4 to scFv-D10 with $(Gly_4Ser)_5$ linker and C-terminal FLAG-6xHis in pET28a(+)	This study

References

1. Datsenko KA & Wanner BL (2000) One-step inactivation of chromosomal genes in *Escherichia coli* K-12 using PCR products. *Proc Natl Acad Sci USA* 97(12):6640-6645.
2. Baba T*, et al.* (2006) Construction of *Escherichia coli* K-12 in-frame, single-gene knockout mutants: the Keio collection. *Mol Syst Biol* 2:2006 0008.
3. Bogsch EG*, et al.* (1998) An essential component of a novel bacterial protein export system with homologues in plastids and mitochondria. *J Biol Chem* 273(29):18003-18006.
4. Guzman LM, Belin D, Carson MJ, & Beckwith J (1995) Tight regulation, modulation, and high-level expression by vectors containing the arabinose P_{BAD} promoter. *J Bacteriol* 177(14):4121-4130.
5. Cherepanov PP & Wackernagel W (1995) Gene disruption in *Escherichia coli*: Tc^R and Km^R cassettes with the option of Flp-catalyzed excision of the antibiotic-resistance determinant. *Gene* 158(1):9-14.
